# From carbon policy to public health: an analysis of pricing decisions in electronics supply chains for costing emissions reduction

**DOI:** 10.3389/fpubh.2026.1723064

**Published:** 2026-03-16

**Authors:** Tianhui Sun, Na Huang, Wanjun Jiang

**Affiliations:** 1 Shanghai Xingjian College, Shanghai, China; 2 College of Economics & Management, Shanghai Ocean University, Shanghai, China; 3 School of Economics and Management, Tongji University, Shanghai, China; 4 School of Management, Shanghai University of Engineering Science, Shanghai, China

**Keywords:** carbon emissions reduction, carbon trading mechanism, electronics supply chain, government oversight, unit health cost loss

## Abstract

Carbon emissions and waste throughout the entire lifecycle of electronic products pose a serious threat to public health. This paper focuses on the synergistic governance perspective of carbon trading and public health, constructs a closed-loop supply chain Stackelberg game model, introduces the “unit health damage cost,” and explores the decision-making evolution mechanism under multiple constraints. Research indicates: (1) The internalization of health costs has dual effects. While it effectively strengthens incentives during periods of low carbon prices, excessive compliance pressure can suppress green investment, resulting in a pronounced inverted *U*-shaped pattern for emission reduction incentives. (2) Price transmission triggers market selection. Compliance costs are passed through to end consumers via prices, marginalizing high-emission enterprises due to price disadvantages and compelling their technological transformation. (3) Structural imbalances in profit distribution occur. Suppliers and retailers bear the primary external cost burden, while recyclers' profits remain decoupled from carbon governance mechanisms. From the perspective of overall social welfare, the public health benefits brought about by the government's emission reduction supervision far outweigh the short-term economic losses of the supply chain, and are conducive to the low-carbon development of the electronic product supply chain and the long-term win-win situation for social welfare. Accordingly, this paper proposes establishing a differentiated policy framework encompassing both compensation for health damages and specialized subsidies for recovery, thereby achieving a win-win outcome for both economic growth and public health.

## 1 Introduction

In recent years, the global climate crisis has spurred strict international regulations like the UN Framework Convention on Climate Change, promoting low-carbon awareness. This consensus stems from both ecological concerns and public health imperatives—the World Health Organization states that air pollution linked to carbon emissions is a leading cause of millions of premature deaths worldwide each year (1). Specifically, elevated PM2.5 levels are significantly associated with increased cardiopulmonary mortality, while ozone exposure drives up medical demand for respiratory diseases. Furthermore, climate-induced heat exposure leads to a surge in emergency department visits and substantial labor productivity losses. In the specific context of manufacturing electronic products, the health risks are compounded, particularly as the artificial intelligence era drives an explosive demand for high-performance computing hardware and accelerated device turnover. Beyond the carbon-related climate effects, improper disposal releases hazardous substances, posing toxicological risks to human health (2). Regarding the scrap and recycling of electronic products, the National Development and Reform Commission estimates that China eliminates 100–120 million used household appliances every year, increasing by an average of 20% per year. Data from the China Household Electrical Appliances Research Institute indicates that in 2024, the total volume of discarded electrical and electronic products recycled reached 220 million units, while the theoretical volume of products reaching end-of-life was approximately 289 million units. However, the recycling industry remains immature. While AI and big data offer tools for optimization (3), relying solely on technical optimization is insufficient. To ensure a sustainable hardware foundation for the AI era, improving pricing and emission reduction strategies by incorporating health externalities is urgent for establishing a green economic system and realizing the “double carbon” goal (4).

At the same time, with the initial investment and gradual improvement of China's carbon trading market, the market-guided low-carbon behavior of enterprises is not necessarily the best low-carbon decision for public welfare, which requires guidance and coordination by the government. In the carbon trading market, the scientific estimation of carbon trading prices in different markets will bring a win-win situation to the government and enterprises. A reasonable corporate carbon tax and environmental regulatory intensity have a synergistic effect on the operational efficiency of the carbon market, which is conducive to promoting carbon emission reduction efforts (5). Fang et al. (6) build an optimization model based on heterogeneous agent technology, and use carbon trading mechanism to reduce carbon emissions according to different market shares of agents. Shajarian et al. (7) studied the dual-channel remanufacturing closed-loop supply chain under carbon footprint and collection competition, and the impact of carbon emission and remanufacturing on the dual-channel forward and reverse logistics. In the new energy vehicle supply chain, government carbon tax strategies may reduce the recycling rate of power batteries, while subsidy strategies can effectively increase the recycling rate (8). Based on the government's carbon tax policy, Bai et al. (9) established a game model of supply chain non-emission reduction, individual emission reduction and cooperative emission reduction, and studied the carbon emission reduction of supply chain under different circumstances and the approval of the government's optimal tax rate. In the process of collaborative emission reduction within the green supply chain, the sum of the collaborative emission reduction benefits and government subsidies is greater than the cost of collaborative emission reduction. Imposing regulatory penalties that exceed the threshold can also influence the “free-rider” behavior of enterprises, thereby promoting faster emission reduction among enterprises in the upstream and downstream of the green supply chain (10).

With the improvement of consumers' quality of life, consumers also begin to pay attention to green consumption. Therefore, in order to win the favor of consumers, enterprises should set supply chain carbon reduction targets and improve their corporate image and competitive advantages. Yang et al. (11) studied the selection of recycling modes in the closed-loop supply chain of remanufacturing under the carbon cap-and-trade policy. In the third-party recycling mode, the total carbon emissions are the least, and different recycling modes of manufacturers will lead to different carbon emissions. Hosseini-Motlagh et al. (12) consider corporate sustainability, based on a reverse supply chain model, and arrive at optimal pricing, sustainability levels, and corporate social responsibility (CSR) decisions under the disruption of decentralized and centralized RSC demand. Kushwaha et al. (13) proposed a mixed integer linear programming model to determine the optimal channel combination for collecting old products from these regions in a reverse supply chain with limited multi-cycles, and the results showed that the timing of implementation of carbon restriction and trade policies has an impact on channel selection of recycled products in multiple regions. En et al. studied the changes in the expected utility of the supply chain and its members brought about by changes in four possibilities of three emission reduction models (carbon emission trading price, consumers' low-carbon awareness, carbon emission and competition from third-party recyclers). The results showed that carbon emission trading price, consumers' low-carbon awareness and carbon emissions were negatively correlated with the expected utility of manufacturers and retailers (14).

Cao et al., (15) in order to solve the decision-making challenges of enterprises under the carbon tax policy and financial constraints, consider a supply chain consisting of manufacturers producing green products and retailers selling these products, and establish five models to study the optimal wholesale price, carbon emission reduction level and order quantity of the two manufacturers, respectively for manufacturers and retailers with or without financial constraints. Mondal et al. (16) studied the competition and cooperation of retailers in the green closed-loop supply chain under the constraints of government intervention and cap-and-trade policies. Liu et al. (17) studied the impact of decision-making patterns on emission reduction decisions when considering carbon taxes, and found that in a centralized decision-making scenario, the cooperation of manufacturers and retailers in reducing emissions is the most advantageous strategy. Considering the retailers' choice of dual channels and pricing in a competitive environment, Jamail et al. (18) studied the impact of wholesale price and greening degree of products on pricing in the supply chain composed of manufacturers and retailers in direct and indirect distribution channels, and showed that vertical cooperation in the supply chain not only increased the profits of the entire supply chain, but also increased the profits of the entire supply chain. It also improves the greenness of the product. Barman et al. (19) used decentralized and centralized models to discuss pricing strategy and green strategy, and compared the optimal decision in all cases to maximize the overall profitability of the supply chain. Based on game theory, Bera et al. studied the long-term dispersion behavior of green supply chain members and the evolutionary stable decision of government intervention in price and sales effort competition among retailers. The results showed that government intervention is conducive to reducing carbon emissions in the production or recycling of products (20). Giri et al. (21) studied the competition in the two-level supply chain based on the duopoly market consisting of two manufacturers and one retailer, considering the leader-follower movement between manufacturers and retailers. Dou et al. (22) found that under the carbon trading mechanism, effective policies are a prerequisite for driving emissions reductions, while successful cooperation within the supply chain depends on the dynamic balance of emissions reduction surpluses, product profitability, and technological maturity.

Most of the current research on carbon reduction in the supply chain focuses on the economic costs of carbon policies, compliance costs, or the incentive effects of reducing emissions in the supply chain. They rarely incorporate the impact of carbon emissions on public health into their studies. In the electronics supply chain, carbon emissions are generated and emitted throughout the production and recycling processes. According to a report by GREENPEACE in 2023, over three-quarters of the carbon emissions in the electronics manufacturing industry come from its supply chain. Moreover, the emission reduction actions of some leading enterprises have been sluggish, failing to meet the goal of the Paris Agreement to limit global warming to 1.5 degrees Celsius by 2030. Excessive carbon emissions exacerbate global warming, causing extreme weather, changes in the spread of infectious diseases, and other problems, which can cause unpredictable damage to human public health. Climate change has also been called the greatest health threat of the 21st century by the World Health Organization. Heat waves directly lead to an increase in mortality rates, while changes in climate patterns facilitate the spread of vector-borne diseases, threatening the health of billions of people worldwide. The report of the International Energy Agency indicates that energy-related carbon emissions are closely linked to severe air health burdens. The supply chain of electronic products, especially the high-energy-consuming processes such as chip manufacturing, component production, and data center operation, are significant contributors to this chain. Therefore, carbon reduction in the electronics supply chain is an important area for addressing climate change, concerning human public health and sustainable development. When studying the issue of carbon reduction in the electronics supply chain and formulating relevant decisions, it is not only necessary to consider carbon emissions as an environmental cost or market factor that needs to be managed, but also to take into account its impact on health. The success of internal cooperation within the supply chain depends on the dynamic balance of emission reduction surplus, product profitability, and technological maturity. This indicates that pricing and profit are the core economic factors driving enterprises' environmental protection decisions. When the government or society expects enterprises to bear the public health damage caused by their carbon emissions, the most effective economic means is to internalize this external cost as an internal cost of the enterprise. This process must be transmitted through prices. Therefore, in this study, the unit health damage cost coefficient was introduced to quantify the damage caused by each unit of carbon emission to public health. Through the Stackelberg game analysis of the pricing decisions in the upstream and downstream of the supply chain, the optimal wholesale price and retail price were determined. In the process of solving the optimal wholesale price and retail price, it was clear that under the constraints of carbon and health, the supply chain achieved the optimal path of balancing economic sustainability and social responsibility. When the decision-making goal shifted from a simple economic-environment trade-off to a tripartite coordination of economy, environment, and health, how the optimal wholesale price, retail price, system profit, and emission reduction changed, and thus establishing a new equilibrium between stimulating deep emission reduction, reasonable distribution of health responsibilities, and maintaining market vitality.

## 2 Model construction and analysis

### 2.1 Problem description

This article considers a closed-loop supply chain for recyclable products consisting of manufacturers, retailers, and recyclers, which takes into account environmental and health externalities. Such products have the dual characteristics of high remanufacturing value and high carbon emissions. On one hand, the metals and components contained in them have a high economic value for recycling; on the other hand, their manufacturing and supply chain processes usually involve intensive energy consumption and carbon emissions. As emissions increase, the accompanying problems can have adverse effects on public health or pose potential threats. Manufacturers, with their core technologies and brand advantages, hold a dominant position in the supply chain and determine the wholesale prices and emission reduction levels of products based on market supply and demand. Retailers, as market followers, set the final retail market prices according to the decisions of manufacturers and the sensitivity of consumers to prices. Recyclers are responsible for the reverse logistics process and determine the recycling prices of waste products based on the market stock of electronic products and the supply and demand functions of the second-hand recycling market.

Hypothesis 1: Suppose that each entity in the electronic product supply chain only sells one type of recyclable electronic product, and both parties are in a situation of information symmetry during the pricing game, meaning that both can fully obtain the strategies adopted by the other party in the pricing process. For the convenience of research, it is assumed that uniform pricing is implemented both online and offline.

Hypothesis 2: Enterprises across the supply chain can collaborate in appropriate ways to jointly reduce carbon emissions, enhance product competitiveness in the market, and achieve long-term sustainable development goals. Recyclers collect waste electronic products from consumers at the specified price, and suppliers collect waste electronic products from recyclers according to the electronic product valuation.

Hypothesis 3: The manufacturing of new electronic products needs to purchase raw materials, so its manufacturing cost is high, the manufacturing cost of recycling and remanufacturing electronic products is small, and the recycled products do not fully participate in the remanufacturing process, there will be part of the residual value. In terms of retail prices, the retail price of new products is generally higher than the price of recycled and remanufactured products.

Hypothesis 4: The demand for electronic products is affected by the retail price, the lower the price, the greater the consumer demand. When there is recycling and remanufacturing of electronic products, it will lead to the transfer of consumers who originally bought new electronic products. At the same time, recycled and remanufactured electronic products and new electronic products are consistent in the use of functions, with a certain degree of substitution, so the price fluctuation of an electronic product will have an impact on the demand for replacement electronic products. Consumer preferences for recycled and remanufactured products also affect demand.

Hypothesis 5: In the operation process of the three-level closed-loop supply chain, the supplier is in the core leadership position, and the retailer is in the following position, and the pricing strategy is adjusted according to the supplier's pricing strategy.

Hypothesis 6: The carbon emissions during the production and recycling of electronic products not only pose environmental pressure but also directly lead to public health issues such as respiratory diseases, thereby generating public health costs resulting from the increase in incidence rates and the rise in medical expenditures. Assuming that the public health economic losses caused by each unit of carbon emissions (such as medical burden, discounted health life loss) are constant. In reality, this is mapped to the generalized health compliance costs, including the increasingly strict pollution fees imposed by various countries on associated pollutants like SOx and NOx, the risk reserves that enterprises pre-emptively set aside to avoid collective lawsuits caused by pollution-related illnesses, and the implicit health governance investments made to meet ESG rating requirements. For instance, the United States and the European Union widely adopt the “social carbon cost” model when formulating climate policies. This model explicitly incorporates “premature deaths and health care expenditures caused by climate change” into the cost calculation basis, meaning that when calculating the economic cost of carbon emissions, health externalities need to be included. In our model, we simplify it to the marginal cost per unit of emission to capture the decision-making behavior of enterprises under the pressure of health accountability. The polluter-pays principle is a core concept in environmental economics, which states that the entity that causes environmental pollution should bear all the costs of pollution control, ecological restoration, and compensation for related damages. Based on the real situation and the “polluter-pays” principle, we set in the model that the supplier, who is at the leading position in the supply chain, should undertake this responsibility to analyze their decision-making behavior under the dual pressures of the environment and health.

### 2.2 Symbol description

Based on the above assumptions and considering the actual composition of the electronics supply chain, we set and describe the relevant parameters of the model. In Table 1, is the quantity of products ordered, that is, *Q*_*n*_ = Q_0_−(*p*_*n*_−*p*_*r*_)/(1−γ). Where Q_0_ is the market capacity and γ is the preference coefficient of consumers for recycled and remanufactured products, and the supplier's carbon emission reduction cost is $\frac{\text{1}}{\text{2}}h{\left(e+e_{u}\right)}^{\text{2}}$ (*h* is the carbon emission reduction cost coefficient). The recycling amount of third-party recyclers is *Q*_*b*_ = *l*+*kp*_*s*_, where *p*_*s*_ is the price of recycling from consumers by third-party recyclers. When the recycling price is 0, the number of electronic products voluntarily recycled by consumers is *l*; *k* represents the consumer's sensitivity to the recycling price. *Q*_*r*_ = (*p*_*n*−_*p*_*r*_)/(1−γ)−*p*_*r*_/γ, represents the unit health loss cost coefficient, which is the cost coefficient of public health damage caused by each unit of carbon emissions. It signifies the public health loss incurred at the societal level for every unit of carbon (or associated pollutants) emitted. *Q*_*r*_ is the supplier's demand for recycled and remanufactured products (*Q*_*b*_>*Q*_*r*_); The government regulates the carbon emission reduction behavior of suppliers. When the carbon emissions of suppliers exceed the carbon quota, they will be punished, and when they are below the carbon quota of the government, they will be rewarded.

**Table 1:** Model parameters and their meanings.

**Symbol**	**Meaning**	**Symbol**	**Meaning**
G	Total carbon quota	γ	Consumer preference coefficient for remanufactured products
*t*	Carbon trading price	*c*_*n*_	Production cost per unit of new product
α	Government supervision factor α≥1	*c*_*r*_	Unit recovery cost of remanufactured products
*e*	Carbon reduction per unit of production	π_*s*_	Supplier profit
*w*_*n*_	Wholesale price of new products	π_r_	Retailer profit
*w*_*r*_	Wholesale prices of recycled and remanufactured products	π_*u*_	Recycler's profit
*p*_*n*_	Retail price of new products	*p*_*s*_	The recycling price of the recycling agency
*p*_*r*_	Retail prices of recycled and remanufactured products	*p*_*b*_	Supplier's recycling price
μ	Additional carbon reduction rate per unit of recycled remanufactured products	π_*s*_	Supplier profit
e_0_	Initial carbon emissions per unit of product	*Q*_*n*_	Quantity of new product orders
*h*	Carbon reduction cost factor	*Q*_*r*_	Orders for recycled and remanufactured products
*k*	Price sensitivity of consumers	*Q*_*b*_	Amount collected by third-party recyclers
*p*_0_	Residual value of recovered products not involved in remanufacturing	*l*	When *p*_*s*_ = 0, the number of electronic products recovered
δ	Unit health loss cost coefficient		

### 2.3 Model construction

**Figure 1:**
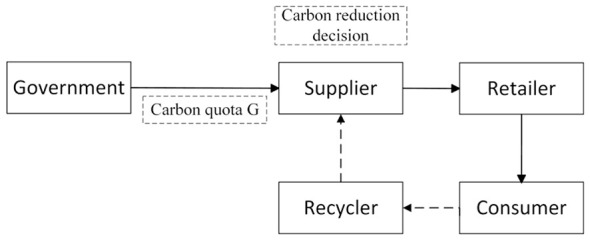
Supply chain carbon emission reduction decision flow chart.

Based on the carbon trading mechanism, this section analyzes the carbon emission reduction decision of suppliers and the pricing decision of suppliers, retailers and recyclers in the supply chain with the participation of the government. Moreover, by analyzing the decision-making flow chart of carbon emission reduction in the supply chain (as shown in Figure 1), the optimal pricing strategy of suppliers, retailers and recyclers under the dual role of the government and the market is studied.

Supplier profit:


$$\begin{array}{l} \pi_{s}\;=(w_{n}-c_{n})Q_{n}+(w_{r}-c_{r})Q_{r}-\frac{1}{2}h(e+\mu e{)}^{2}\\ \;+\;\alpha t[G-(e_{0}-e)Q_{n}-(e_{0}-e-\mu e)Q_{r})]\\ -\;\delta [\left(e_{0}-e)Q_{n}-(e_{0}-e-\mu e)Q_{r}\right)]+(p_{b}-p_{0})Q_{b} \end{array}$$ (1)

Retailer profit:


$$\begin{array}{l} \pi_{r}\;=\left(p_{n}-w_{n}\right)Q_{n}+\left(p_{r}-w_{r}\right)Q_{r} \end{array}$$ (2)

The profits of the third party recyclers are:


$$\begin{array}{l} \pi_{\mu }=\left(p_{b}-p_{s}\right)Q_{b} \end{array}$$ (3)

The Stackelberg game model was constructed to solve and analyze the optimal carbon emission reduction decision and pricing strategy of node enterprises in the supply chain under the government coordination and carbon trading mechanism. The analysis process is as follows:

First of all, the recycler's profit π_μ_ = (*p*_*b*_−*p*_*s*_)*Q*_*b*_ = (*p*_*b*_−*p*_*s*_)(*l*+*kp*_*s*_), take the partial derivative of the recovery price *p*_*s*_ to get $\frac{\partial \pi_{\mu }}{\partial p_{s}}=-l+kp_{b}-2kp_{s}$, and $\frac{\partial^{2}\pi_{\mu }}{\partial p_{s}^{2}}=-2k<0$, then there is an optimal value in the domain of definition, namely:


$$\begin{array}{l} p_{s}=\frac{-l+kp_{b}}{2k} \end{array}$$ (4)

By substituting *Q*_*n*_ = Q_0_−(*p*_*n*_−*p*_*r*_)/(1−γ), *Q*_*r*_ = (*p*_*n*−_*p*_*r*_)/(1−γ)−*p*_*r*_/γ, into (Equation 2), retailer profit can be obtained:


$$\begin{array}{l} \;\pi_{r}\;=\;(p_{n}-w_{n})Q_{n}+(p_{r}-w_{r})Q_{r}=(p_{n}-w_{n})(Q_{0}-\\ \left(p_{n}-p_{r})/(1-\gamma ))+(p_{r}-w_{r})((p_{n}-p_{r})/(1-\gamma )-p_{r}/\gamma \right) \end{array}$$ (5)

Take the derivative of *p*_*n*_ and *p*_*r*_, respectively, and get:


$$\begin{array}{l} \frac{\partial \pi_{r}}{\partial p_{n}}=\frac{2p_{n}-2p_{r}-Q_{0}+\gamma Q_{0}-w_{n}+w_{r}}{-1+\gamma }=0 \end{array}$$ (6)

$$\begin{array}{l} \frac{\partial \pi_{r}}{\partial p_{r}}=\frac{2\gamma p_{n}-2p_{r}-\gamma w_{n}+w_{r}}{\gamma -\gamma^{2}}=0 \end{array}$$ (7)

Set it to 0, get:


$$\begin{array}{l} p_{n}=\frac{1}{2}\left(Q_{0}+w_{n}\right) \end{array}$$ (8)

$$\begin{array}{l} p_{r}=\frac{1}{2}\left(\gamma Q_{0}+w_{r}\right) \end{array}$$ (9)

The supplier profit is:


$$\begin{array}{l} \pi_{s}\;=\;\left(w_{n}-c_{n}\right)\left(Q_{0}-\frac{p_{n}-p_{r}}{\left(1-\gamma \right)}\right)+\left(w_{r}-c_{r}\right)\left(\frac{p_{n}-p_{r}}{1-\gamma }-\frac{p_{r}}{\gamma }\right)-\frac{1}{2}h{\left(e+\mu e\right)}^{2}\\ +\alpha t\left[G-\left(e_{0}-e\right)\left(Q_{0}-\frac{p_{n}-p_{r}}{\left(1-\gamma \right)}\right)-\left(e_{0}-e-\mu e\right)\left(\frac{p_{n}-p_{r}}{1-\gamma }-\frac{p_{r}}{\gamma }\right)\right]\\ -\delta \left[\left(e_{0}-e\right)\left(Q_{0}-\frac{p_{n}-p_{r}}{\left(1-\gamma \right)}\right)-\left(e_{0}-e-\mu e\right)\left(\frac{p_{n}-p_{r}}{1-\gamma }-\frac{p_{r}}{\gamma }\right)\right]+\left(p_{b}-p_{0}\right)\left(l+kp_{s}\right) \end{array}$$ (10)

By substituting *p*_*n*_, *p*_*r*_ and *p*_*s*_, the partial derivatives of *w*_*n*_, *w*_*r*_ and *p*_*b*_ are obtained:


$$\begin{array}{l} \frac{\partial \pi_{s}}{\partial w_{n}}=\frac{e\mu \left(\alpha t+\delta \right)+c_{n}-c_{r}+\left(1-\gamma \right)Q_{0}-2w_{n}+2w_{r}}{2\left(1-\gamma \right)}=0 \end{array}$$ (11)

$$\begin{array}{l} \frac{\partial \pi_{s}}{\partial w_{r}}\\ =\frac{e\left(\alpha t+\delta \right)-e\gamma \left(\alpha t+\delta \right)+e\mu \left(\alpha t+\delta \right)+\gamma c_{n}-c_{r}-e_{0}\left(\alpha t+\delta \right)+\gamma e_{0}\left(\alpha t+\delta \right)-2\gamma w_{n}+2w_{r}}{2\gamma \left(\gamma -1\right)}\\ \;=0 \end{array}$$ (12)

$$\begin{array}{l} \frac{\partial \pi_{s}}{\partial p_{b}}=\frac{1}{2}\left[l+kp_{b}+k\left(p_{b}-p_{0}\right)\right]=0 \end{array}$$ (13)

The optimal pricing strategy is as follows:


$$\begin{array}{l} w_{n}^{*}=\frac{\left(e_{0}-e\right)\left(\alpha t+\delta \right)+Q_{0}+c_{n}}{2} \end{array}$$ (14)

$$\begin{array}{l} w_{r}^{*}=\frac{\left[e_{0}-e\left(1+\mu \right)\right]\left(\alpha t+\delta \right)+c_{r}+\gamma Q_{0}}{2} \end{array}$$ (15)

$$\begin{array}{l} p_{b}^{*}=\frac{kp_{0}-l}{2k} \end{array}$$ (16)

$$\begin{array}{l} p_{s}^{*}=\frac{kp_{0}-3l}{4k} \end{array}$$ (17)

$$\begin{array}{l} p_{n}^{*}=\frac{\left(e_{0}-e\right)\left(\alpha t+\delta \right)+{3Q}_{0}+c_{n}}{4} \end{array}$$ (18)

$$\begin{array}{l} p_{r}^{*}=\frac{\left[e_{0}-e\left(1+\mu \right)\right]\left(\alpha t+\delta \right)+c_{r}+3\gamma Q_{0}}{4} \end{array}$$ (19)

By substituting the profit function, the optimal profit of recyclers, retailers and suppliers can be obtained as follows:


$$\begin{array}{l} \pi_{\mu }^{*}=\frac{{\left(l+kp_{0}\right)}^{2}}{16k} \end{array}$$ (20)

$$\begin{array}{l} \pi_{r}^{*}=\frac{\gamma Q_{0}^{2}}{16}+\frac{{\left[c_{r}-\gamma c_{n}+K\left(e\left(1-\gamma +\mu \right)-e_{0}\left(1-\gamma \right)\right)\right]}^{2}}{16\gamma \left(1-\gamma \right)} \end{array}$$ (21)

$$\begin{array}{l} \pi_{s}^{*}=\frac{{\left(l+kp_{0}\right)}^{2}}{8k}-\frac{\left(l+kp_{0}\right)l}{2k}+\alpha tG-\frac{1}{2}he^{2}{\left(1+\mu \right)}^{2}\\ +\frac{Q_{0}\left[Q_{0}\left(1-\gamma \right)-2\left(c_{n}+K\left(e_{0}-e\right)\right)\right]}{8\left(1-\gamma \right)}+\frac{{\left[c_{r}-\gamma c_{n}+K\left(e\left(1-\gamma +\mu \right)-e_{0}\left(1-\gamma \right)\right)\right]}^{2}}{8\gamma \left(1-\gamma \right)} \end{array}$$ (22)

Under optimal profit conditions,


$$\begin{array}{l} \;\frac{\partial^{2}\pi_{s}^{*}}{\partial e^{2}}\\ =\frac{4h\gamma \left(\gamma -1\right){\left(1+\mu \right)}^{2}+{\left(\alpha t+\delta \right)}^{2}\left[\gamma \left(1+2\mu \right)-{\left(1+\mu \right)}^{2}\right]}{4\gamma \left(\gamma -1\right)}<0 \end{array}$$ (23)

There exists an optimal emission reduction amount, such that $\frac{\partial \pi_{\text{s}}}{\partial e}=0$

The optimal emission reduction is:


$$\begin{array}{l} e^{*}=\frac{\left(\alpha t+\delta \right)\left[c_{r}\left(1-\gamma +\mu \right)-\gamma Q_{0}\left(1-\gamma \right)-\gamma \mu c_{n}+e_{0}\left(\alpha t+\delta \right)\left(1-\gamma \right)\left(1+\mu \right)\right]}{4h\gamma \left[\gamma \left(1+3\mu \right)-{\left(1+\mu \right)}^{2}\right]+{\left(\alpha t+\delta \right)}^{2}\left[{\left(1+\mu \right)}^{2}-\gamma \left(1+2\mu \right)\right]} \end{array}$$ (24)

Lemma 1: The optimal profit of third-party recyclers is independent of government incentive factors, carbon trading prices, and unit health impairment costs, depending solely on the fundamental attributes of the recycling market.

Proof: Based on the optimal decision derived above, we shall now further explore the theoretical mechanism by which key parameters influence supply chain decisions and profits. From the preceding derivation, it follows that the recycler's optimal profit is $\pi_{\mu }^{*}=\frac{{\left(l+kp_{0}\right)}^{2}}{16k}$. Taking partial derivatives with respect to α , *t*, and δ yields $\frac{\partial \pi_{u}^{*}}{\partial \alpha }=0,$, $\frac{\partial \pi_{u}^{*}}{\partial t}=0,$, and $\frac{\partial \pi_{u}^{*}}{\partial \delta }=0,$, respectively. This indicates that pricing for electronic product recycling is primarily driven by the residual value of waste products, rather than being directly linked to carbon emission reduction benefits. Consequently, it occupies a marginal position within traditional carbon trading systems.

Lemma 2: Under the basic profitability condition, both the optimal wholesale price and retail price of new products and remanufactured products increase monotonically with the rise in the generalized environmental regulation cost H.

Proof: Let $p_{n}^{*}=\frac{\left(e_{0}-e\right)\left(\alpha t+\delta \right)+3Q_{0}+c_{n}}{4}$ denote the optimal retail price of the new product. Let *H* = α*t*+δ and H represent the generalized unit environmental regulation cost, encompassing both explicit carbon trading prices and implicit health damage costs. Taking the first derivative of the new product's retail price with respect to H yields $\frac{\partial p_{n}^{*}}{\partial H}=\frac{1}{4}[(e_{0}-e^{*})-H\frac{\partial e^{*}}{\partial H}]$. Let $\left(e_{0}-e^{*}\right)>0$. When the marginal efficiency $\frac{\partial e^{*}}{\partial H}$ of emission reduction technology fails to fully offset direct compliance costs, then $\frac{\partial p_{n}^{*}}{\partial H}>0$ holds. Similarly, $\frac{\partial p_{r}^{*}}{\partial H}>0$ can be proven. As δ and *t* increase, supplier manufacturers tend to pass compliance pressures downstream by raising wholesale prices, ultimately driving up retail prices. High-emission products face steeper price increases due to their greater (*e*_0_−e*), thereby establishing a market screening mechanism. Similarly, it follows that the optimal wholesale prices for new and remanufactured products rise as the generalized environmental regulation cost H increases.

Lemma 3: The optimal carbon reduction quantity does not exhibit a linear increase in response to the generalized environmental regulation cost H, but rather demonstrates diminishing marginal utility and a threshold effect.

Proof: From the optimal reduction quantity formula, it follows that:


$$\begin{array}{l} e^{*}\\ =\frac{\left(\alpha t+\delta \right)\left[c_{r}\left(1-\gamma +\mu \right)-\gamma Q_{0}\left(1-\gamma \right)-\gamma \mu c_{n}+e_{0}\left(\alpha t+\delta \right)\left(1-\gamma \right)\left(1+\mu \right)\right]}{4h\gamma \left[\gamma \left(1+3\mu \right)-{\left(1+\mu \right)}^{2}\right]+{\left(\alpha t+\delta \right)}^{2}\left[{\left(1+\mu \right)}^{2}-\gamma \left(1+2\mu \right)\right]}\\ \;=\frac{H\left[c_{r}\left(1-\gamma +\mu \right)-\gamma Q_{0}\left(1-\gamma \right)-\gamma \mu c_{n}+e_{0}\left(1-\gamma \right)\left(1+\mu \right)H\right]}{4h\gamma \left[\gamma \left(1+3\mu \right)-{\left(1+\mu \right)}^{2}\right]+H^{2}\left[{\left(1+\mu \right)}^{2}-\gamma \left(1+2\mu \right)\right]} \end{array}$$

Let *A* = *c*_*r*_(1−γ+μ)−γ*Q*_0_(1−γ)−γ*μc*_*n*_, *B* = *e*_0_(1−γ)(1+μ), *C* = 4*hγ*[γ(1+3μ)−(1+μ)^2^], *D* = (1+μ)^2^−γ(1+2μ), then the optimal emission reduction can be simplified to $e^{*}=\frac{AH+BH^{2}}{C+DH^{2}}$. Taking the derivative with respect to H reveals that when H is small, the constant term C dominates in the denominator, with $\frac{\partial e^{*}}{\partial H}>0$. The function exhibits near-linear growth, with the emission reduction amount e^*^ increasing rapidly with H. When H is large, *DH*^2^ in the denominator begins to dominate. The growth rate of the denominator in the emission reduction expression will gradually exceed that of the numerator, causing the growth rate of $\frac{\partial e^{*}}{\partial H}$ to slow down or even turn negative. After considering boundary constraints, a decline may even occur. Moreover, due to the non-negative constraints $e^{*}\leq e_{0}$ and $\pi_{s}^{*}\geq 0$, when H becomes excessively high, environmental compliance costs may exceed the threshold acceptable to the enterprise, leading to diminishing marginal returns on emission reduction investments or even a decline in such investments. However, when compliance costs become prohibitively high, the substantial expense forces manufacturers to significantly raise wholesale prices, leading to a sharp decline in downstream market demand. As the total return on green technologies depends on market scale, the marginal benefit of investing in costly emission-reduction technologies diminishes significantly when the market contraction effect caused by high prices outweighs the unit cost savings effect. At this juncture, enterprises rationally shift from proactive emission reduction to passive capacity reduction to mitigate risks, thereby precipitating the abrupt plunge in emission reductions observed in numerical simulations.

## 3 Analysis of numerical examples

The urgency of this research stems from the tangible impact of the electronics industry on the environment and public health. Data from the latest report by the International WEEE Research Alliance reveals that globally, surplus chargers alone from smartphones and IoT devices generate over 11,000 tons of electronic waste and 600,000 tons of carbon emissions annually. Presently, numerous enterprises are engaged in carbon reduction initiatives within the electronics supply chain. This dual threat demands serious attention: carbon emissions exacerbate climate change and pose long-term health risks such as respiratory diseases, while electronic waste directly introduces toxic heavy metals into ecosystems, posing immediate threats to community health through soil and water contamination. Consequently, industry leaders are spearheading the transition toward a low-carbon economy. Taking Apple Inc. as an example, its supply chain accounts for approximately 70% of its total carbon emissions. In 2024, Apple reduced its carbon emissions by about 23 million metric tons, with roughly 2 million metric tons stemming from suppliers' energy efficiency improvements and approximately 21.8 million metric tons from suppliers' clean energy projects. Huawei similarly actively engages its core suppliers in comprehensive emissions reduction planning and implementation. The company routinely conducts annual risk assessments for its primary suppliers, which account for over 90% of its procurement spending, categorizing them into high, medium, and low risk tiers. High- and medium-risk suppliers are included in the annual sustainability audit program. In 2024, 100% of Huawei's top 100 suppliers by procurement value completed the setting of carbon reduction targets.

In the field of electronic product recycling, Dell Technologies (Xiamen City, China) is a renowned manufacturer of computers and electronic equipment, and is also committed to the recycling and reuse of electronic products. Dell offers a variety of recycling programs and services, having recycled over 2.5 billion pounds (1.1 billion kilograms) of used electronic products since 2007. Since 2010, Gree Electric Appliances has established six recycling resource bases in Zhuhai, Changsha, Zhengzhou, Shijiazhuang, Wuhu, and Tianjin. These facilities primarily handle the recovery and processing of discarded electrical and electronic products, end-of-life vehicles, and similar materials. By the end of 2024, Gree Recycling Resources had cumulatively processed over 64.77 million units of various discarded electrical and electronic products, reducing carbon dioxide emissions by 1.0922 million tons. This circular economy model represents not only a commercial approach but also a critical public health intervention preventing hazardous substances from entering the environment. These real-world cases highlight the complex interplay between economic activities, regulatory pressures, and corporate strategies in pursuing a sustainable and healthy future, laying the groundwork for subsequent analysis. Therefore, based on the real-world context and in accordance with the model parameter definitions and assumptions, this paper selects appropriate parameters by referencing practical scenarios and relevant literature. The parameter settings are presented in Table 2. Using MATLAB 2020a, the constructed model analyzes the impact of carbon trading prices and government enforcement factors on carbon emission reductions, wholesale prices, retail prices, and optimal profits. Although the model simultaneously incorporates three variables—government regulation factor α, carbon trading price *t*, and unit health damage cost δ—when analyzing bivariate interactions, we will focus our research on the “market price vs. health cost” trade-off, without reiterating the relationship between “regulation and health.” In the structure of carbon compliance costs, regulatory intensity essentially acts as an “amplifier” for carbon trading prices. The two are highly coupled in mathematical logic, and their transmission pathways to corporate decision-making are isomorphically similar. Therefore, the analysis focuses on the interplay between the explicit “carbon market pricing” and the implicit “social cost of health.” This trade-off not only streamlines the analytical logic but also more intuitively addresses the core theme of this paper: “From Carbon Policy to Public Health.”

**Table 2:** Values of related parameters.

**G**	**Q_0_**	**γ**	***c*_*n*_**	***c*_*r*_**	***p*_0_**	**e_0_**	**μ**	***h***	***k***	***l***
500	400	0.6	200	150	50	0.8	0.8	4,000	0.5	10

### 3.1 Changes in optimal carbon emission reduction

**Figure 2:**
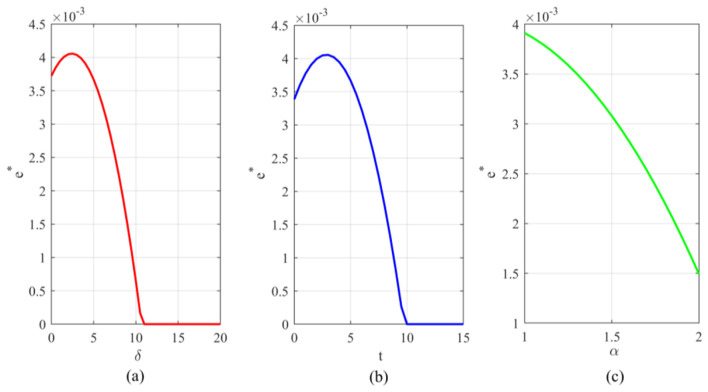
Variation in optimal carbon emission reduction under different parameters: **(a)** Variation in optimal reduction under unit health loss cost; **(b)** Variation in optimal reduction under carbon trading price; **(c)** Variation in optimal reduction under government enforcement factor.

**Figure 3:**
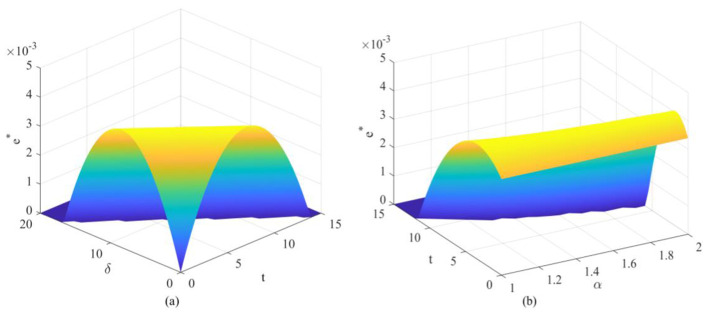
Variation in optimal emission reductions under different parameter combinations: **(a)** Variation in optimal emission reductions under the combined influence of carbon trading price and unit health damage cost; **(b)** Variation in optimal emission reductions under the combined influence of carbon trading price and government enforcement factor.

**Figure 4:**
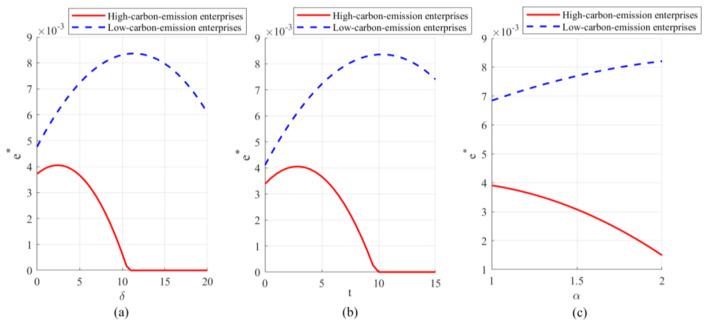
Optimal carbon emission reductions for different enterprises under varying parameters: **(a)** Optimal emission reductions for high-emission and low-emission enterprises under unit health loss cost; **(b)** Optimal emission reductions for high-emission and low-emission enterprises under carbon trading price; **(c)** Optimal emission reductions for high-emission and low-emission enterprises under government enforcement factor.

Observing Figures 2, 3, the optimal emission reduction volume does not increase indefinitely with greater policy intensity but instead exhibits a pronounced inverted *U*-shaped or “initial rise followed by decline” trend. Within the lower range of unit health loss cost, emission reductions rise rapidly as oversight increases. That is, as the health costs and transaction costs of carbon emissions become more apparent, enterprises have strong incentives to invest in emission reduction technologies to lower compliance expenditures. When policy pressure exceeds a certain threshold (e.g., when δ surpasses 10 in Figure 2), a precipitous decline occurs, even falling to zero. This indicates that the cost of emission reduction investments follows a convex function (*he*^2^), while the benefits primarily stem from savings in unit emission costs. When external penalties (α*t*+δ) become excessively high, the optimal strategy for firms may no longer be to stubbornly pursue emissions reductions. Instead, they may opt to significantly reduce production to mitigate risks, or even abandon costly green investments altogether as profit margins are squeezed to zero. This suggests that overly stringent environmental regulations may actually stifle firms' capacity for green innovation, leading to reduced production rather than emissions cuts. Examining the optimal emission reduction levels across firms with different emission profiles (as shown in Figure 4), low-emission firms demonstrate significantly higher reduction activity compared to high-emission counterparts. This highlights the challenge of “turning a large ship around,” reflecting the difficulty for high-emission enterprises to pivot quickly. High-emission enterprises bear a heavy initial emissions burden, resulting in extremely high marginal abatement costs. When facing identical policy shocks, the net benefits they gain from technological upgrades are actually lower than those achieved by low-emission enterprises operating with a lighter emissions load. That is, low-emission enterprises demonstrate greater technological resilience and adaptability during the green transition, while high-emission enterprises are more prone to falling into a “high-cost, low-reduction” lock-in effect.

### 3.2 Changes in optimal wholesale prices

**Figure 5:**
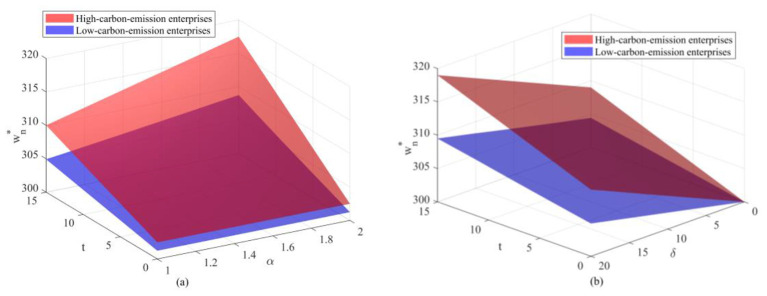
Changes in wholesale prices of new products under different parameter interactions: **(a)** Changes in wholesale prices of new products under the combined effects of carbon trading prices and government enforcement factors; **(b)** Changes in wholesale prices of new products under the combined effects of carbon trading prices and unit health loss costs.

**Figure 6:**
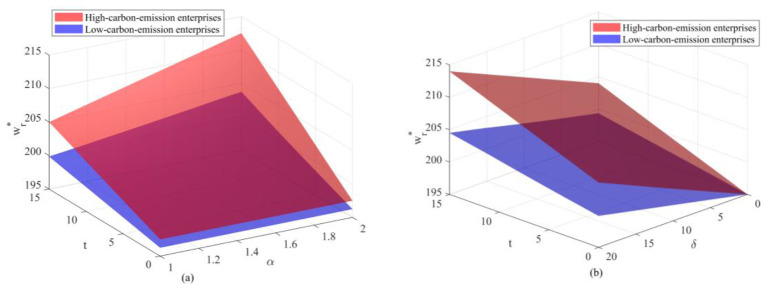
Changes in wholesale prices of remanufactured products under different parameter interactions: **(a)** Changes in wholesale prices of remanufactured products under the combined effects of carbon trading prices and government enforcement factors; **(b)** Changes in wholesale prices of remanufactured products under the combined effects of carbon trading prices and unit health loss costs.

The overall trend from Figures 5, 6 reveals a pronounced “climbing” trajectory in the wholesale price curves for both new and remanufactured products. As government regulations tighten and carbon market prices rise, wholesale prices exhibit a non-linear upward shift. Under dual policy pressures, suppliers—as the direct entities responsible for carbon emissions—face increasingly high compliance costs. To maintain marginal profits, suppliers must internalize these environmental costs and pass them downstream, ultimately manifesting as rising wholesale prices for both new and remanufactured products. Comparing high-emission enterprises with low-emission enterprises reveals a clear stratification phenomenon. The red curve consistently hovers above the blue curve, with the vertical gap between them widening as government enforcement intensifies. This phenomenon clearly demonstrates that enterprises with higher initial carbon emissions exhibit greater sensitivity to policy shocks. For high-emission enterprises, every unit increase in carbon trading prices or regulatory tightening translates into heavier cost burdens, compelling them to set higher wholesale prices to hedge risks. Conversely, low-emission enterprises leverage technological advantages to gain greater pricing flexibility and competitive edge. The three-dimensional surfaces representing new product wholesale prices and remanufactured product wholesale prices exhibit striking similarities in shape, trajectory, and even curvature. From the perspective of the pricing formula structure, both are heavily driven by the core carbon cost factor (α*t*+δ). Since carbon policies exert pressure on the production side in the same direction and with consistent structure, the response functions of the two product prices to policy variables exhibit a high degree of structural isomorphism. However, beneath the similar “form” lies a different “substance.” The two products exhibit a significant difference in their *Z*-axis intercepts—constrained by higher raw material procurement and processing costs (*c*_*n*_), the benchmark price of the new product consistently exceeds that of the remanufactured product. This phenomenon of “trend consistency with varying price levels” precisely demonstrates the model's robustness in handling multi-product pricing logic: the policy environment determines the direction of price fluctuations, while production costs dictate the absolute price levels.

### 3.3 Changes in optimal retail prices

**Figure 7:**
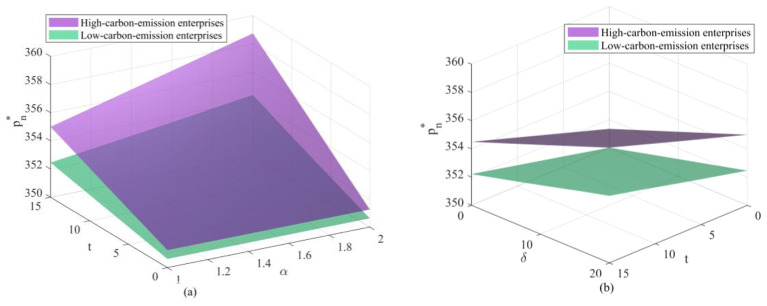
Changes in retailers' new product retail prices under different parameter interactions: **(a)** Changes in new product retail prices under the combined effects of carbon trading prices and government enforcement factors; **(b)** Changes in new product retail prices under the combined effects of carbon trading prices and unit health loss costs.

**Figure 8:**
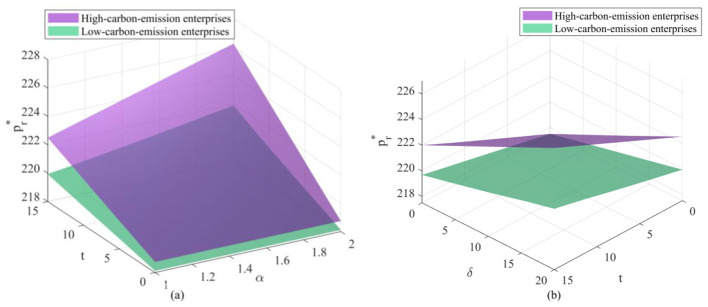
Changes in the retailer's remanufactured product retail price under different parameter interactions: **(a)** Changes in the remanufactured product retail price under the combined effects of carbon trading price and government enforcement factor; **(b)** Changes in the remanufactured product retail price under the combined effects of carbon trading price and unit health loss cost.

As shown in the changes depicted in Figure 7 (retail price of new products) and Figure 8 (remanufactured retail price), retail prices exhibit a positive correlation with carbon trading prices, unit health damage costs, and regulatory intensity. The monotonically increasing trend of the surface indicates that retailers, as rational market entities, do not absorb wholesale price increases passed down from upstream suppliers on their own. Instead, they pass on the “carbon costs” and “health compensation” to consumers by raising end-user prices. This implies that within the electronics supply chain, consumers may ultimately bear the costs of environmental governance. While this transmission of price signals increases the purchasing burden on consumers, it holds macroeconomic rationale: leveraging prices to curb demand for high-carbon products, thereby compelling green behavioral shifts at the consumer end. In retail price comparisons, high-emission enterprises consistently command significantly higher prices than their low-emission counterparts. This price differential constitutes a market penalty for high-emission enterprises. High-emission enterprises will lose price competitiveness in end markets as they must set higher prices to cover their substantial compliance costs. This price gap will widen further as the cost per unit of health damage increases—reflecting society's growing emphasis on public health. This will trigger a positive market selection mechanism: price-sensitive consumers will naturally gravitate toward low-emission enterprises' affordable green products, thereby leveraging market forces to accelerate the elimination or transformation of high-carbon enterprises. Comparing the trend shifts between new products and remanufactured products, while both follow a consistent overall trajectory, the price baseline for new products remains consistently higher than that for remanufactured products. This indicates that even under extreme carbon policy pressure, the market positioning of new products as the premium option and remanufactured products as the high-value-for-money alternative has not fundamentally shifted. Changes in carbon policies have primarily raised the industry's overall “water level” without disrupting the internal hierarchical structure of products.

### 3.4 Variations in optimal profit

**Figure 9:**
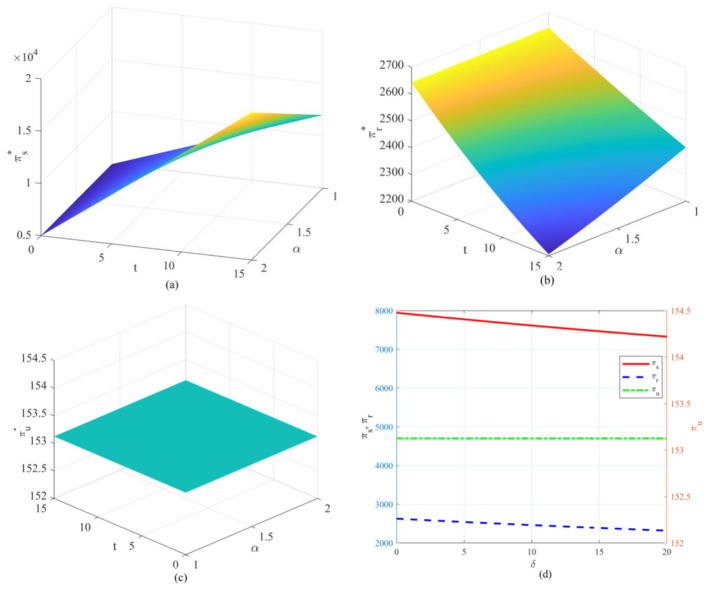
Variation in optimal profit under different parameter interactions: **(a)** variation in supplier optimal profit under the combined effect of carbon trading price and government incentives; **(b)** variation in retailer optimal profit under the combined effect of carbon trading price and government incentives; **(c)** variation in recycler optimal profit under the combined effect of carbon trading price and government incentives; **(d)** variation in optimal profit for suppliers, retailers, and recyclers under the unit health loss cost.

Observing the two-dimensional graph (Figure 9), the recycler's profit curve stands in stark contrast to the other two parties. It appears as a horizontal straight line in the two-dimensional plot and as a completely flat plane in the three-dimensional plot. Regardless of how stringent government regulations become, how volatile carbon trading prices fluctuate, or how high the unit health damage cost rises, recyclers' profits appear to possess a form of immunity, remaining consistently stable at a fixed level. This reveals a key characteristic within the current model architecture—the decoupling between reverse logistics and the carbon trading mechanism. In the Stackelberg game framework, recyclers' profits primarily depend on the residual value of recycled products not participating in remanufacturing, consumer price sensitivity, and recycling volumes—rather than being directly linked to carbon emission reduction revenue distribution. In other words, the pressure from carbon trading and health compensation is largely absorbed within the forward logistics chain, failing to effectively penetrate the reverse recycling segment. This also suggests that relying solely on raising carbon prices or imposing health taxes cannot automatically stimulate market vitality at the recycling end. This disconnect in policy transmission serves as a warning: when designing circular economy policies, we must establish independent incentive mechanisms for the recycling segment. Otherwise, recyclers will remain permanently excluded from policy dividends, making it difficult to achieve full-chain green synergy. As the cost per unit of health damage caused by carbon emissions increases, both supplier and retailer profits exhibit a monotonically decreasing trend. This indicates that without external subsidies, making implicit health costs explicit will squeeze the profit margins of core enterprises in the supply chain. Although suppliers can shift some pressure by raising wholesale prices, this transfer is not lossless—increased end-user prices suppress market demand, leading to a scenario where both volume and price decline simultaneously. Suppliers, in particular, bear the steepest decline in their profit curve as the primary entities responsible for emissions reduction investments and health compensation, enduring the most significant policy-induced pain.

### 3.5 Social welfare and consumer surplus analysis

To comprehensively assess the social external effects of incorporating public health costs into supply chain decisions, this section introduces consumer surplus (CS) and social welfare (SW) as measurement indicators. Based on the model assumptions, the demand functions for the construction and analysis part are established and analyzed. Consumer surplus is defined as the integral of the difference between the consumers' willingness to pay for new products and remanufactured products and the actual payment price. According to the consumers' demands and product preferences in the assumptions, the calculation formula for consumer surplus is as follows:


$$\begin{array}{l} CS\;=\;\int_{Q_{0}-Q_{n}}^{Q_{0}}\left(v-p_{n}^{*}\right)dv\;+\;\int_{Q_{0}-Q_{n}-Q_{r}}^{Q_{0}-Q_{n}}\left(\gamma v-p_{r}^{*}\right)dv \end{array}$$ (25)

Among them, *v* represents the consumer's evaluation of the utility of a unit product, reflecting the additional economic satisfaction that the consumer gains during the purchasing process.

Social welfare (SW) is composed of the total profit of the supply chain system, consumer surplus, government carbon trading revenue and expenditures, as well as the total public health damages. Since the model has set that suppliers bear the cost of health externalities, according to the Coase Theorem, this cost has been internalized through the “polluter pays” principle. Therefore, social welfare can be defined as the total utility of the entire society minus production costs and actual damages:


$$\begin{array}{l} SW=\Pi_{SC}+CS+\alpha t\left(G-E_{total}\right)+\delta E_{total} \end{array}$$ (26)

**Figure 10:**
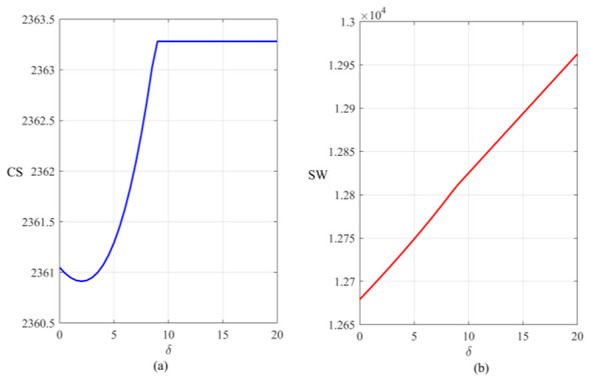
Changes in consumer surplus and social welfare under the influence of the unit health loss cost coefficient: **(a)** Changes in consumer surplus under the influence of the unit health loss cost coefficient; **(b)** Changes in social welfare under the influence of the unit health loss cost coefficient.

Based on the simulation results (Figure 10), it can be analyzed that regarding the relationship between consumer surplus and unit health cost, as δ changes, consumer surplus shows a trend of decreasing—increasing—stabilizing. This indicates that in the initial stage of the introduction of the carbon trading policy, the carbon reduction technologies of enterprises were not yet mature and the unit health loss cost was relatively low. Enterprises lacked sufficient motivation to invest in expensive carbon reduction technologies. They chose to bear the unit health cost and mainly converted it into compliance burdens and passed them on to the final retail prices, resulting in a decline in consumer surplus. As the cost of health deterioration keeps increasing beyond the critical threshold, the high external costs force manufacturers to invest in emission reduction technologies. The emission reduction volume has significantly increased, and the decline in the unit compliance cost brought about by the emission reduction effect exceeds its own growth rate. The environmental compliance cost per unit of product has decreased significantly, verifying the innovation compensation effect in Porter's hypothesis. This technological dividend effectively offsets costs, leading to a decline in prices at the consumer end and an increase in consumer surplus. As unit health costs continue to rise, the curve eventually reaches a plateau, indicating that this corresponds to the upper limit of the emission reduction level in the model. Regardless of how δ continues to increase, it no longer has a marginal impact on the cost structure of enterprises, and the terminal retail price and consumer surplus are locked at the optimal steady-state level. In the right chart, social welfare shows a stable and monotonically increasing trend with respect to δ. This indicates that although there was a brief loss of consumer economic surplus during the initial stage of policy implementation, the public health dividends brought about by emission reduction have dominated the welfare composition. As δ continues to increase, although the nominal costs of enterprises increase, the value of potential health damage reduction brought about by technological upgrades shows a linear growth. This suggests that by reasonably setting the parameter of unit health damage and internalizing externalities, it is possible to effectively break the zero-sum game between economic development and public health, promote the transition of social welfare from high-carbon inefficiency to zero-carbon efficiency, and achieve comprehensive Pareto improvement of the economy-environment-health.

## 4 Conclusion

This paper constructs a closed-loop supply chain game model involving manufacturers, retailers, and third-party recyclers. It innovatively introduces the key variable of “unit health impairment cost” to thoroughly examine optimal decision-making and profit distribution within the electronic supply chain under the triple constraints of government regulation, carbon trading mechanisms, and public health externalities. Through theoretical derivation and numerical simulation, the following core conclusions and management implications are drawn.

First, internalizing public health costs serves as a powerful engine driving green innovation, yet it exhibits a threshold effect. Research indicates that incorporating public health damage costs significantly boosts corporate motivation for emissions reduction compared to carbon trading policies alone. This is particularly true during periods of low carbon prices, where the rigid constraints imposed by health costs effectively supplement regulatory frameworks. However, emissions reduction incentives do not increase indefinitely with policy intensity; instead, they follow a pronounced inverted *U*-shaped pattern. When government oversight and per-unit health damage costs exceed a certain threshold, the surge in marginal compliance costs causes firms to shift from proactive emissions reduction to passive production cuts, potentially crowding out green investments. This implies that when formulating carbon policies integrated with public health, governments should avoid “shock therapy.” Instead, they must precisely identify the policy equilibrium point where incentives align to prevent excessive pressure from undermining supply chain resilience.

Second, the price transmission mechanism creates a market screening effect for high-carbon emitting enterprises. The model indicates that carbon compliance costs and per-unit health damage costs are ultimately partially passed on to end consumers through increases in wholesale and retail prices. In this process, high-carbon emitting enterprises, burdened by heavier historical emissions legacies, exhibit significantly higher price sensitivity than low-carbon enterprises. This results in their products facing greater price disadvantages in the end market. This policy-driven price differential effectively establishes a positive market selection mechanism—leveraging consumers' pricing power to accelerate the elimination of technologically outdated, high-energy-consuming production capacity, thereby compelling enterprises to transition toward low-carbon technologies. Supplier enterprises should also proactively invest in forward-looking emissions reduction initiatives, lowering initial emission intensity ahead of mandatory policy enforcement. By voluntarily assuming health responsibilities, companies can not only mitigate future potential liability risks but also capture price-sensitive market share through lower end-user pricing, thereby achieving the “good money drives out bad money” effect.

Third, the profit distribution within the electronics supply chain exhibits structural imbalances, urgently requiring a differentiated compensation mechanism. Following the introduction of unit health impairment costs, a trade-off exists between the supply chain's economic and social benefits in the short term. Manufacturers and retailers, as primary actors in forward logistics, directly bear the economic costs of internalizing externalities, resulting in significantly compressed profit margins. Conversely, recyclers operating in reverse logistics demonstrate “immunity” to fluctuations in carbon trading prices and unit health loss cost coefficient, revealing a disconnect between recycling operations and carbon governance mechanisms. This indicates that relying solely on penalty or tax mechanisms cannot automatically achieve incentives across the entire chain. To prevent recyclers from being left behind in the green transition, supply chain manufacturers should establish internal transfer payment mechanisms based on extended producer responsibility systems, channeling a portion of emission reduction benefits back to recyclers. Governments should create recycling subsidies separate from carbon trading to address profit imbalances caused by externalities transmission bottlenecks, ensuring every link in the closed-loop supply chain remains profitable and incentivized to pursue voluntary emission reductions.

Fourth, from the perspective of overall social welfare, the public health benefits brought about by government regulation and carbon governance offset the economic costs. Simulation results show that initially, due to enterprises passing on compliance costs to the final prices, there will indeed be a short-term decline in consumer surplus. However, as enterprises improve their emission reduction technologies and the unit compliance costs decrease, the welfare level of consumers will gradually recover. The total social welfare always shows an upward trend as the health cost loss coefficient increases. This indicates that the value of public health losses reduced by government regulation is much higher than the economic efficiency lost in the supply chain due to compliance. In the future, when formulating regulatory policies, the government should not only focus on short-term market price fluctuations, but also attach importance to the long-term social value of improving public health, so as to achieve a win-win situation for economic development and social welfare.

Achieving the green transformation of electronic supply chains and improving public health in the future cannot rely solely on market carbon price signals. Instead, it requires building a multidimensional policy framework encompassing “targeted regulation, health-based pricing, market screening, and differentiated subsidies.” This also offers a new perspective for subsequent research on quantifying specific health subsidy ratios.

## Data Availability

The original contributions presented in the study are included in the article/supplementary material, further inquiries can be directed to the corresponding author.
